# *In vitro* and *in vivo* evaluation of the genotoxic and antigenotoxic potential of the major Chios mastic water constituents

**DOI:** 10.1038/s41598-018-29810-y

**Published:** 2018-08-15

**Authors:** Elena Drosopoulou, Dimitris Vlastos, Ioanna Efthimiou, Paraskevi Kyrizaki, Sofia Tsamadou, Maria Anagnostopoulou, Danai Kofidou, Maxim Gavriilidis, Despoina Mademtzoglou, Penelope Mavragani-Tsipidou

**Affiliations:** 1 Department of Genetics, Development and Molecular Biology, School of Biology, Faculty of Science, Aristotle University of Thessaloniki, Thessaloniki, Greece; 2 Department of Environmental and Natural Resources Management, University of Patras, Agrinio, Greece

## Abstract

Chios mastic products are well-known for their broad applications in food industry, cosmetics, and healthcare since the antiquity. Given our recent finding that Chios mastic water (CMW) exerts antigenotoxic action, in the present study, we evaluated the genotoxic as well as the antigenotoxic potential of the four major compounds of CMW, namely, verbenone, α-terpineol, linalool, and trans-pinocarveol. The cytokinesis block micronucleus (CBMN) assay in cultured human lymphocytes and the *Drosophila* Somatic Mutation And Recombination Test (SMART), also known as the wing spot test, were employed. None of the four major CMW’s constituents or their mixtures showed genotoxic or recombinogenic activity in either of the assays used. Co-treatment of each of the constituents with MMC revealed that all except trans-pinocarveol exerted antigenotoxic potential. Moreover, co-administration of verbenone with linalool or α-terpineol presented statistically significant reduction of MMC-induced mutagenicity. In conclusion, the major CMW constituents were shown to be free of genotoxic effects, while some exerted antigenotoxic activity either alone or in combinations, suggesting synergistic phenomena. Our results provide evidence on the key antigenotoxicity effectors of the plant extract CMW.

## Introduction

Chios Mastic gum, a natural product of protected designation of origin, is derived from the endemic bush *Pistacia lentiscus* (L.) var. *chia* (Duham) in the Greek island of Chios^1^. Mastic products are meeting international demand due to the multitude of beneficial properties that are attributed to them since the antiquity and they are widely used in the selfcare, food, and cosmetics sectors worldwide^2,3^. Their beneficial biological activities have been thoroughly documented by a number of studies showing their antibacterial, antimicrobial, anti-inflammatory, antioxidant, antiatherogenic, and anticancer properties. The above properties are extensively covered by the recent “assessment report on *Pistacia lentiscus* L. resin” of the European Medicines Agency^3^.

Despite the increasing international interest for mastic products and their proposed clinical applications^3^, no data on their potential genotoxicity are available with the exception of our recent studies on the genotoxicity and antigenotoxicity status of the commercially available Chios Mastic oil (CMO), the essential oil of mastic resin, and Chios Mastic Water (CMW), the aqueous solution produced during the steam distillation of mastic resin. CMO was found to lack genotoxic, mutagenic or recombinogenic activity, while CMW not only did not exert any genotoxic activity but also showed antimutagenic action against the DNA damage induced by mitomycin-C (MMC)^4,5^.

In an effort to identify which CMW constituents exert protective effects against the mutagenic effects of MMC, in the present study, we evaluated the genotoxic and antigenotoxic activity of its major components, namely verbenone, α-terpineol, linalool, and trans-pinocarveol^6^. We further assessed the genotoxic and antigenotoxic potential of mixtures of these components to explore possible synergistic/antagonistic phenomena.

The cytokinesis block micronucleus (CBMN) assay, a simple yet sensitive *in vitro* assay was applied in human lymphocytes for the investigation of the potential genotoxic, antigenotoxic and cytotoxic effects of the compounds^7^. Micronuclei (MN) are formed as a result of the inability of acentric chromosome fragments or whole chromosomes to migrate to the poles during the anaphase stage, which renders it possible to detect aneugenic and clastogenic effects in cells having undergone cell division after being exposed to the test chemical^7,8^.

The genotoxic and antigenotoxic potential properties of the tested authentic compounds or mixtures of compounds were further assessed with the somatic mutation and recombination test (SMART). This *in vivo* assay allows the detection of mutagenic, recombinogenic, and antigenotoxic effects of substances in *Drosophila melanogaster* (Meigen)^9,10^, an animal model with numerous advantages for mutation research and genetic toxicology, such as the extensive knowledge of its genetics, the ease of its laboratory maintenance and genetic manipulations and the high homology between fly and human genes^11–14^. In this assay, the standard (ST) cross is employed to detect the mutagenic activity of the tested substances, while the high-bioactivation (HB) cross characterized by high levels of cytochrome P450-dependent bioactivation capacity, is used for the detection of promutagens and procarcinogens^9,15^.

The genotoxic and antigenotoxic assessment of the main constituents of the CMW of the present study are expected (i) to strengthen the safety status of the tested authentic constituents, (ii) to reveal the compounds that are behind the beneficial properties of mastic water, and (iii) to explore possible synergistic or antagonistic activity of the used mixtures. Taking into account that both mutagenesis and recombination are intimately related to cancer^16,17^, the above information could contribute to exploiting protective biological agents in the primary prevention of mutation-related diseases.

## Results

### Genotoxicity and antigenotoxicity tested with CBMN assay

**Figure 1:**
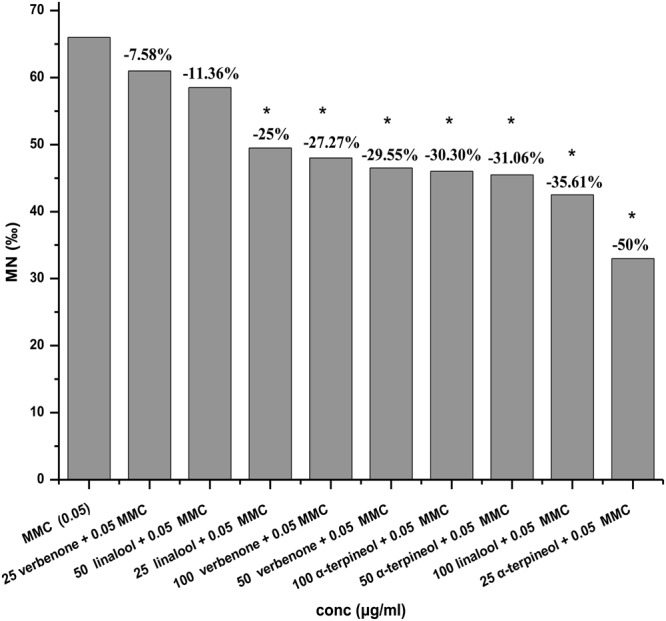
Reduction (%) of MN frequency induced by MMC (0.05 *μ*g/ml) in the presence of different concentrations (25, 50, and 100 *μ*g/ml) of verbenone, α-terpineol and linalool. *Significant difference compared to MMC.

The CBMN assay was applied to evaluate the genotoxic activity of the four CMW constituents at three concentrations (25, 50 and 100 *μ*g/ml), and the same doses were tested combined with MMC in order to identify the antigenotoxic effect of the constituents against the genotoxic damage induced by MMC. None of the constituents at any dose tested induced MN formation compared to the control (Table 1) indicating absence of genotoxic activity. Treatment with 0.05 *μ*g/ml of MMC provoked a statistically significant increase in MN and micronucleated binucleated (BNMN) cell frequencies as expected. A significant decrease in MN frequencies was observed for three of the four constituents i.e. verbenone, linalool, and α-terpineol, when they were given along with MMC (Table 1). Specifically, all α-terpineol concentrations paired with MMC induced statistically significant decrease of the MN frequencies (*p* < 0.001, *p* < 0.01, *p* < 0.01), as did the two highest verbenone concentrations (*p* < 0.01, *p* < 0.05), and the lowest and highest linalool concentrations (*p* < 0.05, *p* < 0.01) (Fig. 1). α-terpineol demonstrated the highest antigenotoxic activity leading to a 50% decrease of the genotoxicity at its lowest concentration. Trans-pinocarveol did not demonstrate any antigenotoxic potential at the lowest concentration. The two other concentrations were found to be extremely toxic for the cells and the slides were not scorable for MN due to the low numbers of binucleated (BN) cells. In summary, all concentrations of the four constituents used in the present study were not genotoxic themselves, while verbenone, linalool and α-terpineol reduced the genotoxic effect of MMC.

**Table 1:** Frequencies of BNMN and MN as well as CBPI values in cultured human lymphocytes treated with verbenone, α-terpineol, linalool, trans-pinocarveol alone or combined with mitomycin-C (MMC).

Concentration (*μ*g/ml)	BNMN	MN	CBPI
0	5.5 ± 1.5	5.5 ± 1.5	1.76 ± 0.04
verbenone			
25	3.0 ± 0.0	3.0 ± 0.0	1.72 ± 0.02^1^
50	2.5 ± 0.5	2.5 ± 0.5	1.60 ± 0.03^2^
100	3.5 ± 0.5	3.5 ± 0.5	1.58 ± 0.06^2^
α-terpineol			
25	2.5 ± 0.5	2.5 ± 0.5	1.68 ± 0.10^2^
50	3.0 ± 1.0	3.0 ± 1.0	1.66 ± 0.03^2^
100	2.5 ± 0.5	2.5 ± 0.5	1.59 ± 0.05^2^
linalool			
25	3.0 ± 1.0	3.0 ± 1.0	1.74 ± 0.06^2^
50	3.5 ± 0.5	3.5 ± 0.5	1.64 ± 0.00^2^
100	4.5 ± 1.5	4.5 ± 1.5	1.59 ± 0.03^2^
trans-pinocarveol			
25	4.5 ± 1.5	4.5 ± 1.5	1.35 ± 0.04^2^
50	2.5 ± 0.5	2.5 ± 0.5	1.34 ± 0.09^2^
100	3.0 ± 0.0	3.5 ± 0.5	1.33 ± 0.01^2^
MMC			
0.05	65.5 ± 4.5^2^	66.0 ± 5.0^2^	1.52 ± 0.04^2^
verbenone + MMC			
25 + 0.05	58.5 ± 2.5^2^	61.0 ± 1.0^2^	1.38 ± 0.02^2,c^
50 + 0.05	45.5 ± 8.5^2,b^	46.5 ± 8.5^2,b^	1.45 ± 0.05^2^
100 + 0.05	45.0 ± 7.0^2,b^	48.0 ± 10.0^2,a^	1.32 ± 0.04^2,c^
α-terpineol + MMC			
25 + 0.05	32.5 ± 4.5^2,c^	33.0 ± 5.0^2,c^	1.34 ± 0.01^2,c^
50 + 0.05	45.0 ± 1.0^2,b^	45.5 ± 0.5^2,b^	1.33 ± 0.04^2,c^
100 + 0.05	45.0 ± 2.0^2,b^	46.0 ± 2.0^2,b^	1.29 ± 0.02^2,c^
linalool + MMC			
25 + 0.05	48.0 ± 0.0^2,a^	49.5 ± 0.5^2,a^	1.35 ± 0.04^2,c^
50 + 0.05	56.0 ± 4.0^2^	58.5 ± 1.5^2^	1.31 ± 0.02^2,c^
100 + 0.05	41.0 ± 2.0^2,c^	42.5 ± 1.5^2,b^	1.42 ± 0.06^2,b^
trans-pinocarveol + MMC			
25 + 0.05	63.0 ± 1.0^2^	65.0 ± 1.0^2^	1.19 ± 0.01^2,c^
50 + 0.05	—	—	1.09 ± 0.01^2,c^
100 + 0.05	—	—	1.07 ± 0.01^2,c^

BN: binucleated cells; BNMN: micronucleated binucleated cells; MN: micronuclei; CBPI: Cytokinesis Block Proliferation Index; MMC: Mitomycin-C; MF (‰) ± se, mean frequencies (‰) ± standard error; MN were scored in 2000 binucleated lymphocytes per experimental point;

^1,2^Significant difference compared to control at p < 0.01, and p < 0.001, respectively.

^a,b,c^Significant difference compared to MMC at p < 0.05, p < 0.01, and p < 0.001 respectively; G-test for BNMN and MN; χ^2^ for CBPI.

All four constituents were further tested for cytotoxicity with and without MMC by the determination of the Cytokinesis Block Proliferation Index (CBPI). The CBPI presented statistically significant decrease at all the constituents’ concentrations with or without MMC. The cytotoxicity was most pronounced in trans-pinocarveol (Table 1). The cytotoxity, in terms of % cytostasis, did not exceed 55 ± 5% in any case, according to the OECD guideline^7^.

### Genotoxicity and antigenotoxicity tested with SMART

The major CMW constituents, verbenone, α-terpineol, linalool, and trans-pinocarveol were further tested, by applying the SMART Test, for possible genotoxic and recombinogenic activity, at the concentration of 0.025 or 0.05 *μ*g/ml resembling the content of each constituent in CMW. Τhe concentration of 2.5 *μ*g/ml was also tested for comparison with previous data using the same assay^18^. A parallel experiment using acetone solution (0.75%) was carried out as the negative control, since all compounds were dissolved in 0.75% acetone before use. The results together with the negative control experiment for ST and BH crosses are summarized in Table 2. The comparative screening for spontaneous and induced mutagenesis showed that none of the tested substances exerted genotoxic or recombinogenic effects in the ST or HB crosses at the doses used in the present study (Table 2). Although at the ST cross the statistical test lead to inconclusive results in some cases, they were interpreted as having minimal biological significance, since the wing spot frequencies were close to the ones of the negative control.

**Table 2:** Frequency of mutations (mosaic spots/wing) for each spot category (small, large, twin, and total) in *D. melanogaster* treated with Chios mastic water constituents (concentrations in *μ*g/ml) alone or combined with mitomycin-C (MMC) in the Standard (ST) and High Bioactivation (HB) crosses.

Concentration (*μ*g/ml)	Number of wings	Frequency of spots per wing and diagnosis^1^			
		Small single spots	Large single spots	Twin spots	Total spots
ST cross					
0	50	0.16 (8)	0.06 (3)	0.00 (0)	0.22 (11)
verbenone					
0.05	50	0.22 (11) i	0.02 (1)−	0.02 (1) i	0.26 (13) i
2.5	50	0.16 (8) i	0.00 (0)−	0.02 (1) i	0.18 (9)−
α-terpineol					
0.05	50	0.14 (7) i	0.00 (0)−	0.02 (1) i	0.16 (8) −
2.5	50	0.20 (10) i	0.00 (0)−	0.00 (0) i	0.20 (10) i
linalool					
0.025	50	0.20 (10) i	0.06 (3) i	0.02 (1) i	0.28 (14) i
2.5	50	0.12 (6) i	0.04 (2)−	0.00 (0) i	0.16 (8)−
trans-pinocarveol					
0.025	50	0.30 (15) i	0.06 (3) i	0.04 (2) i	0.40 (20) i
2.5	50	0.22 (11) i	0.00 (0)−	0.04 (2) i	0.26 (13) i
MMC					
2.5	50	0.54 (27)+	0.34 (17) +	0.16 (8) +	1.04 (52)+
verbenone + MMC					
0.05 + 2.5	50	0.38 (19)+	0.30 (15)+	0.24 (12)+	0.92 (46)+
2.5 + 2.5	50	0.36 (18)+	0.20 (10)+	0.00 (0) i *	0.56 (28)+*
α-terpineol + MMC					
0.05 + 2.5	50	0.36 (18)+	0.14 (7) i *	0.10 (5)+	0.60 (30)+
2.5 + 2.5	50	0.48 (24)+	0.14 (7) i *	0.06 (3) i	0.68 (34)+
linalool + MMC					
0.025 + 2.5	50	0.42 (21)+	0.42 (21)+	0.10 (5)+	0.94 (27)+
2.5 + 2.5	50	0.42 (21)+	0.28 (14)+	0.06 (3) i	0.76 (38)+
trans-pinocarveol + MMC					
0.025 + 2.5	50	0.70 (35)+	0.30 (15)+	0.14 (7)+	1.14 (57)+
2.5 + 2.5	50	0.52 (26)+	0.20 (10)+	0.06 (3) i	0.78 (39)+
HB cross					
0	50	0.36 (18)	0.06 (3)	0.04 (2)	0.46 (23)
verbenone					
0.05	50	0.40 (20)−	0.06 (3) i	0.00 (0) −	0.46 (23)−
2.5	50	0.48 (24) i	0.04 (2)−	0.02 (1) i	0.54 (27)−
α-terpineol					
0.05	50	0.36 (18)−	0.06 (3) i	0.02 (1) i	0.44 (22)−
2.5	50	0.36 (18)−	0.10 (5) i	0.02 (1) i	0.48 (24) −
linalool					
0.025	50	0.48 (24) i	0.00 (0)−	0.02 (1) i	0.50 (25)−
2.5	50	0.26 (13) −	0.00 (0)−	0.00 (0)−	0.26 (13)−
trans-pinocarveol					
0	50	0.36 (18)−	0.00 (0)−	0.02 (1) i	0.38 (19)−
2.5	50	0.30 (15)−	0.06 (3) i	0.00 (0)−	0.36 (18)−
MMC					
2.5	50	0.86 (43)+	0.72 (36)+	0.26 (13) +	1.84 (92)+
verbenone + MMC					
0.05 + 2.5	50	0.92 (46)+	0.52 (26)+	0.36 (18)+	1.80 (90)+
2.5 + 2.5	50	0.62 (31)+	0.28 (14) + *	0.14 (7) i	1.04 (52) + *
α-terpineol + MMC					
0.05 + 2.5	50	0.54 (27) i	0.44 (22) + *	0.20 (10)+	1.18 (59)+
2.5 + 2.5	50	0.60 (30) i	0.34 (17) + *	0.10 (5) i	1.04 (52) + *
linalool + MMC					
0.025 + 2.5	50	0.88 (44)+	0.62 (31)+	0.26 (13)+	1.76 (88)+
2.5 + 2.5	50	0.80 (40) +	0.98 (49)+	0.52 (26)+	2.30 (115)+
trans-pinocarveol + MMC					
0.025 + 2.5	50	0.84 (42)+	0.82 (41)+	0.28 (14)+	1.94 (97)+
2.5 + 2.5	50	0.84 (42)+	0.68 (34)+	0.56 (28)+	2.08 (104)+

^1^The number of mutant spots is given in parenthesis. Symbols next to values signify the following: +, positive mutagenic effect;−, no mutagenic effect; i, inconclusive effect (p = 0.05); Statistical diagnosis according to Frei and Würgler^50^.

The antimutagenic effect of verbenone, α-terpineol, linalool and trans-pinocarveol against MMC-induced genotoxic damage was evaluated by the simultaneous administration of MMC with the above used doses of each constituent. MMC was used at final concentration of 2.50 *μ*g/ml since this concentration has previously been shown to be mutagenic in our system^4,5^ and, thus, it also served as positive control. As expected, this well-known mutagen significantly increased all wing spot categories in both ST and HB crosses in comparison to the negative control (Table 2). After co-treatment of MMC with each tested compound, a reduction of the induced total or/and individual wing spot frequency was observed, in most cases. However, this reduction was found to be statistically significant only for verbenone and α-terpineol. Specifically, verbenone, at the concentration of 2.50 *μ*g/ml, significantly reduced tween (*p* = 0.01) and total (*p* = 0.034) wing spots in the ST cross, and large (*p* = 0.004) and total (*p* = 0.017) ones in the HB cross (Table 2). α-terpineol significantly reduced large wing spots at both concentrations in both crosses (*p* *=* 0.012–0.046); however the total wing spot reduction was found to be statistically significant (*p* = 0.014)only at the HB cross at the high concentration.

Based on the evident antigenotoxic activity of verbenone in both ST and HB *D. melanogaster* crosses, we decided to treat larvae with combinations of this compound with any of the other three main CMW constituents. Furthermore, we supplied all four substances simultaneously. As shown in Table 3, none of these combinations was genotoxic at any concentration tested. Similarly to the results of the individual compounds, all treatments were clearly negative in the HB cross, while in the ST cross some mixtures presented inconclusive results with minimal biological significance. At the HB cross, when all compounds were simultaneously administered at levels close to the naturally occurring in CMW (i.e. 0.025–0.05 *μ*g/ml), not only did they yield less mutant/recombinant clones than any compound alone, but they resulted in a 56% spot reduction compared to the negative control (Table 3).

**Table 3:** Frequency of mutations (mosaic spots/wing) for spot each category (small, large, twin, and total) in *D. melanogaster* treated with mixtures of Chios mastic water constituents (concentrations in alone or combined with mitomycin-C (MMC) (2.5 *μ*g/ml) in the Standard (ST) and High Bioactivation (HB) crosses.

Concentration (*μ*g/ml)	Number of wings	Frequency of spots per wing and diagnosis^1^			
		Small single spots	Large single spots	Twin spots	Total spots
ST Cross					
0	50	0.16 (8)	0.06 (3)	0.00 (0)	0.22 (11)
verbenone + α-terpineol					
0.05 + 0.05	50	0.24 (12) i	0.00 (0)−	0.08 (4) i	0.32 (16) i
2.5 + 2.5	50	0.10 (5)−	0.02 (1)−	0.00 (0) i	0.12 (6)−
verbenone + linalool					
0.05 + 0.025	50	0.16 (8) i	0.00 (0)−	0.00 (0) i	0.16 (8) −
2.5 + 2.5	50	0.20 (10) i	0.00 (0)−	0.04 (2) i	0.24 (12) i
verbenone + trans-pinocarveol					
0.05 + 0.025	50	0.12 (6) i	0.00 (0)−	0.00 (0) i	0.12 (6)−
2.5 + 2.5	50	0.12 (6) i	0.14 (7) i	0.02 (1) i	0.28 (14) i
verbenone + α-terpineol + linalool + trans-pinocarveol					
0.05 + 0.05 + 0.025 + 0.025	50	0.38 (19)+	0.00 (0)−	0.00 (0) i	0.38 (19) i
2.5 + 2.5 + 2.5 + 2.5	50	0.14 (7) i	0.08 (4) i	0.00 (0) i	0.22 (11) i
MMC					
2.5	50	0.54 (27)+	0.34 (17)+	0.16 (8)+	1.04 (52)+
verbenone + α-terpineol + MMC					
0.05 + 0.05 + 2.5	50	0.50 (25) +	0.72 (36)+	0.14 (7)+	1.36 (68)+
2.5 + 2.5 + 2.5	50	0.54 (27)+	0.10 (5) i	0.10 (5)+	0.74 (37)+
verbenone + linalool + MMC					
0.05 + 0.025 + 2.5	50	0.26 (13) i	0.32 (16)+	0.14 (7)+	0.72 (36)+
2.5 + 2.5 + 2.5	50	0.28 (14) i	0.08 (4) i *	0.00 (0) i *	0.36 (18) i *
verbenone + trans-pinocarveol + MMC					
0.05 + 0.025 + 2.5	50	0.36 (18)+	0.28 (14)+	0.14 (7)+	0.78 (39)+
2.5 + 2.5 + 2.5	50	0.48 (24)+	0.62 (31)+	0.20 (10)+	1.30 (65)+
verbenone + terpineol + linalool + trans-pinocarveol + MMC					
0.05 + 0.05 + 0.025 + 0.025 + 2.5	50	0.78 (39)+	0.50 (25)+	0.12 (6)+	1.40 (70)+
2.5 + 2.5 + 2.5 + 2.5 + 2.5	50	0.52 (26)+	0.26 (13)+	0.14 (7) +	0.92 (46)+
HB Cross					
0	50	0.36 (18)	0.06 (3)	0.04 (2)	0.46 (23)
verbenone + α-terpineol					
0.05 + 0.05	50	0.40 (20)−	0.06 (3) i	0.04 (2) i	0.50 (25)−
2.5 + 2.5	50	0.30 (15)−	0.00 (0)−	0.00 (0)−	0.30 (15)−
verbenone + linalool					
0.05 + 0.025	50	0.20 (10)−	0.10 (5) i	0.04 (2) i	0.34 (17)−
2.5 + 2.5	50	0.30 (15)−	0.02 (1)−	0.04 (2) i	0.36 (18)−
verbenone + trans-pinocarveol					
0.05 + 0.025	50	0.44 (22) i	0.06 (3) i	0.00 (0)−	0.50 (25)−
2.5 + 2.5	50	0.24 (12)−	0.00 (0)−	0.06 (3) i	0.30 (15)−
verbenone + α-terpineol + linalool + trans-pinocarveol					
0.05 + 0.05 + 0.025 + 0.025	50	0.20 (10)−	0.00 (0)−	0.00 (0)−	0.20 (10)−
2.5 + 2.5 + 2.5 + 2.5	50	0.10 (5)−	0.24 (12)+	0.02 (1) i	0.36 (18)−
MMC					
2.5	50	0.86 (43)+	0.72 (36)+	0.26 (13)+	1.84 (92)+
verbenone + α-terpineol + MMC					
0.05 + 0.05 + 2.5	50	0.50 (25) i	0.26 (13) + *	0.06 (3) i *	0.82 (41) + *
2.5 + 2.5 + 2.5	50	0.64 (32)+	0.48 (24) +	0.16 (8) i	1.28 (64)+
verbenone + linalool + MMC					
0.05 + 0.025 + 2.5	50	0.12 (6) + *	0.30 (15) + *	0.00 (0) − *	0.42 (21)− *
2.5 + 2.5 + 2.5	50	0.14 (7) + *	0.12 (6) i *	0.02 (1) i *	0.28 (14)− *
verbenone + trans-pinocarveol + MMC					
0.05 + 0.025 + 2.5	50	0.82 (41)+	0.56 (28)+	0.18 (9)+	1.56 (44)+
2.5 + 2.5 + 2.5	50	0.90 (45)+	0.82 (41)+	0.32 (16)+	2.04 (102)+
verbenone + terpineol + linalool + trans-pinocarveol + MMC					
0.05 + 0.05 + 0.025 + 0.025 + 2.5	50	0.30 (15)−	0.54 (27)+	0.14 (7) i	0.98 (49)+*
2.5 + 2.5 + 2.5 + 2.5 + 2.5	50	0.10 (5)+*	0.24 (12)+*	0.02 (1) i *	0.36 (18)− *

^1^The number of mutant spots is given in parenthesis. Symbols next to values signify the following: + , positive mutagenic effect; −, no mutagenic effect; i, inconclusive effect (p = 0.05); Statistical diagnosis according to Frei and Würgler^50^.

In an effort to further understand the role of the major CMW constituents in the antigenotoxic profile of CMW, the above mentioned combinations of the CMW constituents were evaluated combined with 2.50 *μ*g/ml MMC. At the ST cross (Table 3), co-administration of the mixtures with MMC resulted in total spot decrease in many cases, with the mixture of verbenone + linalool being the most effective. At the HB cross, a more profound reduction of the mutant clones was observed in almost all combinations tested reaching statistical significance for the mixtures of (i) verbenone + α-terpineol at the low concentration (ii) verbenone + linaloolat both concentrations, and (iii) all four compounds at both concentrations (Table 3). Similarly to the ST cross, the mixtures of verbenone + linalool showed the highest antigenotoxic activity against MMC reaching the negative control mutation rates in both concentrations. Total spot frequency lower than the one of the negative control was also observed for the mixture of all compounds at the high concentration used (Table 3).

## Discussion

Mastic products have been shown to possess a variety of biological activities and to hold therapeutic promise^3^. As part of our ongoing research on the genotoxic and antigenotoxic potential of natural products we recently identified CMW as a mastic extract with antigenotoxic properties^4^. The complexity of such extracts makes it difficult to identify the specific components that exert DNA-protecting effects, since this activity is often the result of additive, synergistic or antagonistic effects of major and/or minor constituents^19^. Thus, in the present study we explore the safety status (as evidenced by lack of genotoxicity) along with the antigenotoxic and cytotoxic potential of the main CMW constituents alone and in different combinations, applying both the *in vitro* CBMN assay and the *in vivo* SMART test.

None of the four CMW constituents or their mixtures was found to be genotoxic, mutagenic or recombinogenic, in our testing systems and under our experimental conditions. More specifically, they did not induce increased MN frequencies in cultured human lymphocytes as revealed by the use of the CBMN assay (Table 1). Furthermore, the SMART test demonstrated that the frequency of mutagenic events stayed close to the one of the negative control even at the HB cross, which better detects promutagens and procarcinogens^15^ (Table 2). Our results are in accordance with several lines of evidence confirming the lack of genotoxicity and mutagenicity of α-terpineol and linalool in different bacterial, yeast, insect and mammalian systems^18,20–22^. Given the close association of mutagenesis with cancer^17^, it is also considered relevant that neither α-terpineol nor linalool were found to induce any increase in pulmonary tumors in a susceptible mouse strain^23^. For verbenone and trans-pinocarveol as well as for mixtures of the tested compounds no data is available. To our knowledge, the present study is the first evaluation of their genotoxic potential.

Given the absence of genotoxic activity of the above CMW authentic constituents or mixtures, their potential antigenotoxic activity against the mutagenic agent MMC was assessed as well. MMC is an antitumor, antibiotic compound with a range of genotoxic effects including the inhibition of DNA synthesis, cross-linking complementary DNA strands, mutagenesis and clastogenesis^24^. It was found to be genotoxic in all *in vitro* and *in vivo* test systems in mammalian cells and animals and was clearly demonstrated as carcinogenic agent^25^. In agreement with previous reports^4,5,25^, MMC was found to be mutagenic in our assays, as well (Tables 1–3). In *Drosophila*, MMC had a stronger effect in the strain with high constitutive levels of cytochrome P450 (i.e. HB cross) (Tables 2 and 3), which is in line with the fact that CYP450-dependent activities are involved in its metabolic activation^26^.

Among the tested CMW constituents, verbenone was found to exert antigenotoxic activity in the human lymphocyte as well as in both (ST and HB) crosses of the *Drosophila* test, α-terpineol in the CBMN and in the ΗΒ cross of the SMART assay, while linalool exerted antigenotoxic potential only in the CBMN assay. Trans-pinocarveol did not show any antigenotoxic activity (Fig. 1, Tables 1 and 2). The antigenotoxic potential of linalool has been previously tested leading, however, to contradictory results depending on the assay and the DNA damage-inducing agents used^20,22,27^. On the other hand, no data are available on verbenone, α-terpineol and trans-pinocarveol, although many beneficial properties are attributed to them^6,28^. Hence, our results constitute the first evidence for the antigenotoxic activity of verbenone and α-terpineol.

The antigenotoxic activity of verbenone and α-terpineol found in the present study (Fig. 1, Tables 1 and 2) could suggest the potential implication of these constituents in the previously shown antigenotoxic activity of CMW^4^. Generally, the major compounds of complex mixtures reflect quite well the properties of the extract; however, the modulatory effects of minor components or the synergism of molecules are not to be underestimated. To evaluate the potential synergistic, additive or antagonistic effects among the four CMW constituents tested here, we assessed the antigenotoxic activity of combinations of verbenone with each or all of the other major CMW ingredients by the SMART assay. Indeed, under our experimental conditions, some of the mixtures had stronger antigenotoxic effects than each compound alone (Table 3). Although in most cases the effect was not higher (i.e. synergy) or equal (i.e. additive effect) to the sum of the individual effects, our results clearly show that the combination of verbenone and linalool present the highest antigenotoxic activity suggesting synergistic phenomena. Our results are in accordance with previous data showing that linalool participates in synergistic interactions with other monoterpenes^29^.

Based on our data, it is challenging to define the exact mechanism(s) behind the antigenotoxic activity that some CMW constituents alone or in combinations exert and further work is required for determining their protective effect. Suggested mechanisms include (i) inhibition of penetration of mutagens into the cells, (ii) direct inactivation of mutagens by scavenging, (iii) inhibition of metabolic conversion by CYP450 of promutagens into mutagens, (iv) reduction of direct DNA-clastogen interaction, (v) activation of detoxification, (vi) interference with DNA repair systems, and (vii) cytotoxicity increase or decrease^19,30,31^. In support to the first case, electron microscopy has shown that linalool and α-terpineol alter membrane permeability and function^29^.

Apart from the antigenotoxic activity all constituents at all tested concentrations exerted cytotoxicity as revealed by the significant decrease of CBPI values in the CBMN assay (Table 1). Our results are supported by literature data demonstrating that verbenone, α-terpineol, and linalool exhibit cytotoxic activity^32–34^. No studies have been conducted in reference to the cytotoxicity of trans-pinocarveol. Considering the close relationship between DNA damage and cancer development, the combination of antigenotoxic with cytotoxic activities of the mastic water constituents would suggest their potential anti-cancer properties and application in anticancer medicinal treatments. Indeed, several mastic extracts or mastic constituents have been shown to exert anti-cancer activities, such as reduced proliferation (cells)/growth (tumors), increased apoptosis, blockage in G1 phase of the cell cycle, and suppressed NF- κB activity, *in vitro* and *in vivo* against different tumors or cancer cells^35–45^.

In conclusion, the safety status of the main components of CMW, verbenone, α-terpineol, linalool, and trans-pinocarveol –either alone or in combination– was established here, as evidenced by the lack of genotoxic effects in the CBMN and SMART assays. Moreover, we were able to identify some biologically active components (e.g. verbenone and α-terpineol), or components’ mixtures (verbenone with linalool), which could account for the observed antigenotoxic activity of CMW against the MMC-induced DNA damage^4^. Noteworthy the antigenotoxic action was more profound following co-administration of verbenone and linalool indicating synergistic effects among them. Establishing active naturally occurring compounds or extracts that counteract DNA damage and genomic mutation is hoped to have a therapeutic prospect in the prevention of mutation-related diseases, such as genetic disorders, carcinogenicity, and aging.

## Methods

### Chemicals

The tested compounds were commercially supplied (Sigma Chemical Co, St Louis, MO, USA). Their purity is as follows: verbenone (≥99%), (+)-α-Terpineol (≥97%), linalool (≥99%), and (−)-trans-Pinocarveol (≥97%). Mitomycin-C (MMC) and cytochalasin-B (Cyt-B) were also purchased from Sigma (St. Louis, MO, USA). Ham’s F-10 medium, foetal bovine serum and phytohaemaglutinin were commercially supplied (Gibco, UK). Faure’s solution was prepared by mixing 100 g distilled H_2_O, 100 g chloral hydrate (C_2_H_3_Cl_3_O_2_), 40 g glycerine (C_3_H_8_O_3_) and 60 g arabic gum. All other chemicals and solvents were of the highest grade commercially available. Stocks of the compounds and solutions were stored at 4 °C until use.

### Ethical approval and informed consent

The study was approved by the Ethical Committee of the University of Patras and performed in accordance with relevant guidelines and regulations. Informed consent was obtained from all participants and/or their legal guardian/s.

### CBMN assay in human lymphocytes *in vitro*

The CBMN assay was performed according to the standard procedure and criteria proposed by OECD^7^ and described by Vlastos *et al*.^4,5^.

After informed consent blood samples were obtained from two healthy, nonsmoking male individuals (less than 30 years) who were not exposed to radiation, drug treatment or any viral infection in the recent past, according to their declaration. Whole blood (0.5 ml) was added to 6.5 ml of Ham’s F-10 medium containing 1.5 ml of fetal bovine serum and 0.3 ml of phytohaemagglutinin to stimulate cell division. All four CMW constituents were diluted in ethanol before being added to the culture medium. They were added at three different doses (25, 50 and 100 *μ*g/ml) alone or in combination with MMC (0.05 *μ*g/ml), 24 h after culture initiation. After 44 h of incubation, cytochalasin-B (final concentration 6 *μ*g/ml) was added to the cultures to block cytokinesis of dividing cells. Cultures were incubated at 37 °C in a humidified atmosphere of 5% CO_2_ for 72 h. 72 h after the initiation of culture, cells were harvested and collected by centrifugation. A mild hypotonic treatment with 3:1 solution of Ham’s medium and milli-q H_2_O was left for 3 min at room temperature which was followed by 10 min fixation (for at least 3 times) with a fresh 5:1 solution of methanol/acetic acid. Cells were stained for 10 min with 7% Giemsa. In total, 2000 binucleated (BN) cells with preserved cytoplasm were scored per experimental point to calculate the MN frequency according to standard criteria^46,47^.

To determine possible cytotoxic effects, the cytokinesis block proliferation index (CBPI) was evaluated by counting at least 1000 cells for each experimental point (500 cells per culture of each donor) as previously described^48^. CBPI is given by the equation: CBPI = [M1 + 2M2 + 3(M3 + M4)]/N, where M1, M2, M3 and M4 correspond to the numbers of cells with one, two, three and four nuclei and N is the total number of cells.

### Somatic Mutation and Recombination Test

Three *Drosophila* stocks carrying visible wing genetic markers on the third chromosome (kindly provided by Dr. Spano, Laboratory of Mutagenesis, Institute Of Genetics and Biochemistry, Federal University of Uberlandia, Uberlandia, Brazil) were used: (i) the *mwh* strain (with genetic constitution *y; mwh j*), which contains the wing cell marker *multiple wing hair* (*mwh*), (ii) the *flr*^*3*^strain (with genetic constitution *flr*^3^*/In (3LR)TM3, ri p*^*p*^*sep l(3)89Aa bx*^34*e*^ *e Bd*^*s*^*)*,which contains the wing cell marker *flare*^3^ (*flr*^3^), and (iii) the *ORR* strain (with genetic constitution *ORR; flr*^3^*/In (3LR)TM3, ri p*^*p*^*sep l(3)89Aa bx*^34*e*^ *e Bd*^*s*^), that has chromosomes 1 and 2 from DDT-resistant Oregon R(R) line, which are responsible for a high constitutive level of cytochrome P(CYP)6A2^49^. Two crosses were used: (i) Standard (ST) cross (virgin females of *flr*^3^ strain crossed with *mwh* males)^9,10^ and (ii) High Bioactivation (HB) cross (virgin females of *ORR* strain with *mwh* males)^15^. The latter cross improves the performance of the wing SMART in the case of promutagens activated via cytochrome P450-dependent metabolic pathways^49^. Insects were maintained at 24 ± 1 °C and 60% RH, at a photoperiod 16:8 (light:dark) on a yeast–glucose medium.

The experiments were carried out as described by Graf *et al*.^10,11^ with slight modifications. Briefly, eggs were collected during a six-hour period in culture bottles and 72 ± 3 hours after laying, series of 40 larvae were transferred to treatment vials containing 0.85 g of *Drosophila* Instant Medium (Carolina Biological Supply, Burlington, NC, USA) rehydrated with 4 ml of the tested solutions. Larvae were subjected to chronic feeding on these culture media for the rest of their larval life (approximately 48 hours). The hatched adults were selected and stored in 70% v/v ethanol:glycerol (1:1, v/v). Both crosses produced two types of progeny, easily distinguished by the BdS marker: (i) marker-heterozygous flies (mwh +/+ flr^3^) with phenotypically wild-type wings and (ii) balancer-heterozygous flies (mwh/TM3, BdS) with phenotypically serrate wings. The wings of the trans-heterozygous (mwh +/+ flr^3^) were removed, mounted in Faure’s solution and scored at 400x magnification for the presence of mosaic spots. The observed spots were grouped into four categories based on the size, number, and type of cells showing malformed wing hairs as: (i) small single spots (with one or two affected cells, either *mwh* or *flr*^3^), (ii) large single spots (with three or more affected cells, either *mwh* or *flr*^3^), (iii) twin spots (consisting of both *mwh* and *flr*^*3*^subclones), and (iv) total spots^9^. Ten replicates per treatment were performed. Since no considerable difference in survival rates of hatched flies from independent experiments was observed, 50 wing samples per treatment were randomly selected for analysis. A total of 3400 wings were scored in this study.

### Statistical analysis

The results of the CBMN assay are expressed as the mean frequency ± standard error (MF ± se). The statistical analysis of the MN data was accomplished using the G-test for independence on 2 × 2 tables, whereas the chi-square test (*χ*^*2*^ test) was used for the analysis of CBPI among each treatment. Differences at p < 0.05 were considered significant. The Origin 7.0 (Origin Lab Corporation, Northampton, MA, USA), the Minitab statistical software (Minitab Inc., PA, USA) and the Statistical Package for Social Sciences (SPSS) for Windows, version 17 were the statistical software used for data analysis.

SMART assay genotoxicity results were analyzed using the multiple-decision procedure, which is based on the conditional binomial test and the chi-squared test (K. Pearson’s criterion), as previously described^4,5^. Antigenotoxicity results were analyzed using the nonparametric Mann-Whitney U-test to compare the spot frequencies in pairs (negative control versus compounds; MMC versus MMC + compounds)^4,5^. The used significance level was 5%.

## Data Availability

All data generated or analysed during this study are included in this published article.
